# Perturbation of nucleotide metabolism - the driving force of oncogene-induced senescence

**DOI:** 10.18632/oncotarget.1023

**Published:** 2013-05-08

**Authors:** Zbigniew Darzynkiewicz

**Affiliations:** Brander Cancer Research Institute and Department of Pathology, New York Medical College, Valhalla, NY. USA

Induction of premature (stress induced) cellular senescence of normal cells provides the mechanism eliminating proliferative capability of potentially carcinogenic cells and thereby is considered to be a barrier preventing neoplastic tranformation [1]. On the other hand, induction of senescence in tumor cells provides a therapeutic opportunity to treat cancers that may be potentially susceptible [2]. Among factors inducing senescence are oxidative DNA damage leading to persistent replication stress, activation of oncogenes and loss of tumor suppressor genes [1]. It was recently reported however that perturbation of the nucleotide metabolism can be added into these already “classic” inducers. The early observations by Mannava et al.,[3]and Liu et al [4], established evidence of a cause-effect relationship between C-MYC, the oncogene that is capable of suppressing senescence and nucleotide metabolism. The authors reported that downregulation of the C-MYC in several human metastatic melanoma lines via lentivirus-based shRNA led to repression of several genes encoding enzymes related to dNTP metabolism such as thymidylate synthase (TS), inosine monophosphate dehydrogenase 2 (IMPDH2) and phosphoribosyl pyrophosphate synthetase 2 (PRPS2). This observation provided a rationale to explore a potential link between dNTP metabolism, DNA damage and the oncogene induced senescence (OIS).

Additional evidence on the role of perturbed dNTP pools in induction of DNA damage and replication stress was provided by Bester et al.,[5]. Their studies were focused on oncogenes regulating cell cycle progression, specifically the Rb-E2F pathway. The authors observed that aberrant activation of this pathway by HPV-16 E6/E7 or cyclin E oncogenes led to a reduction of cellular nucleotide levels. Interestingly, when C-MYC was coexpressed with cyclin E, its presence led to an increase in transcription of nucleotide biosynthesis genes, prevented the reduction of nucleotide pool and rescued the replication-stress induced DNA damage [5].

In the subsequent studies Mannava et al.,[6] provided direct evidence that down-regulation of nucleoside pools is a cause of DNA damage and senescence in normal human fibroblasts (NHFs) overexpressing HRAS^G12V^, a classical model of oncogene-induced senescence. Among the enzymes down-regulated by activated HRAS were TS, ribonucleotide reductase subunits M1 and M2 (RRM1 and RRM2). Ectopic coexpression of TS, RRM1 and RRM2 or addition of deoxyribonucleosides to a large extent prevented DNA damage, decreased expression of senescence-associated phenotypes as well as attenuated the extent of proliferation arrest. Interestingly, overexpression of TS, RRM1 and RRM2 in quiescent NHFs had no effect on their proliferation arrest suggesting that unlike the OIS, quiescence is not induced by perturbation of dNTP pools. Moreover, in a separate study, Mannava et al.,[7]demonstrated that shRNA-mediated depletion of RRM2 and TS was sufficient to induce senescence in melanoma cells. The observation that knockdown of RRM2 and TS in cells with ctivated oncogenes drives them to senescence provides further evidence that abundance of dNTPs promotes melanoma by suppressing cellular senescence.

Most of the above findings were reproduced in a study of Aird et al.,[8] on a role of suppressed nucleotide pools in DNA damage and oncogene-induced senescence. Additionally the authors reported that RRM2 repression correlated with senescent status of melanocytes in human benign nevi and melanoma harboring oncogenic BRAF or NRAS. Furthermore, high expression of RRM2 correlates with poor survival of melanoma patiens[8].

The seminal findings on different cellular and oncogene models that perturbation of dNTP pools leads to DNA damage, replication stress, irreversible senescence [5-8],may have important clinical implications. The example that with similar oncogene activation the senescence of melanocytes induced by shortage of dNTPs results in nevi remaining benign for years whereas abundance of dNTPs leads to malignant melanoma, opens new possibilities to explore potential role of dNTPs as the target in cancer treatment. In light of these findings the commonly used anticancer drug hydroxyurea which targets dNTPs or 5-fluorouracil that affects thymidylates should be revaluated in terms of its possible senescence-promoting efficiency. For years, acceptance of antitumor drugs by the FDA was based on response criteria assessed by tumor shrinkage that most likely occurs as a result of rapid cell death by apoptosis. Assessment of the tumor response by its size reduction criteria precluded the detection of potential treatment benefits that could be mediated by induction of senescence of tumor cells which may remain alive but reproductively dead. It is tempting to explore whether a combined treatment of cancer initially by cytotoxic regimen, relying on induction of apoptosis, be followed by prolonged treatment promoting senescence of cancer cells, is more effective than the traditional chemotherapy.
